# *In Vitro* and *In Vivo* Activity of a Palladacycle Complex on *Leishmania (Leishmania) amazonensis*

**DOI:** 10.1371/journal.pntd.0001626

**Published:** 2012-05-15

**Authors:** Carolina de Siqueira Paladi, Isabella Aparecida Salerno Pimentel, Simone Katz, Rodrigo L. O. R. Cunha, Wagner Alves de Souza Judice, Antonio C. F. Caires, Clara Lúcia Barbiéri

**Affiliations:** 1 Departamento de Microbiologia, Imunologia e Parasitologia, Universidade Federal de São Paulo, São Paulo, São Paulo, Brazil; 2 Centro de Ciências Naturais e Humanas, Universidade Federal do ABC, Santo André, São Paulo, Brazil; 3 Centro Interdisciplinar de Investigação Bioquímica, Universidade de Mogi das Cruzes, Mogi das Cruzes, São Paulo, Brazil; McGill University, Canada

## Abstract

**Background:**

Antitumor cyclopalladated complexes with low toxicity to laboratory animals have shown leishmanicidal effect. These findings stimulated us to test the leishmanicidal property of one palladacycle compound called DPPE 1.2 on *Leishmania (Leishmania) amazonensis*, an agent of simple and diffuse forms of cutaneous leishmaniasis in the Amazon region, Brazil.

**Methodology/Principal Findings:**

Promastigotes of *L. (L.) amazonensis* and infected bone marrow-derived macrophages were treated with different concentrations of DPPE 1.2. In *in vivo* assays foot lesions of *L. (L.) amazonensis*-infected BALB/c mice were injected subcutaneously with DPPE 1.2 and control animals received either Glucantime or PBS. The effect of DPPE 1.2 on cathepsin B activity of *L. (L.) amazonensis* amastigotes was assayed spectrofluorometrically by use of fluorogenic substrates. The main findings were: 1) axenic *L. (L.) amazonensis* promastigotes were destroyed by nanomolar concentrations of DPPE 1.2 (IC50 = 2.13 nM); 2) intracellular parasites were killed by DPPE 1.2 (IC50 = 128.35 nM), and the drug displayed 10-fold less toxicity to macrophages (CC50 = 1,267 nM); 3) one month after intralesional injection of DPPE 1.2 infected BALB/c mice showed a significant decrease of foot lesion size and a reduction of 97% of parasite burdens when compared to controls that received PBS; 4) DPPE 1.2 inhibited the cysteine protease activity of *L. (L.) amazonensis* amastigotes and more significantly the cathepsin B activity.

**Conclusions/Significance:**

The present results demonstrated that DPPE 1.2 can destroy *L. (L.) amazonensis in vitro* and *in vivo* at concentrations that are non toxic to the host. We believe these findings support the potential use of DPPE 1.2 as an alternative choice for the chemotherapy of leishmaniasis.

## Author Summary

Leishmaniasis is an important public health problem with an estimated annual incidence of 1.5 million of new human cases of cutaneous leishmaniasis and 500,000 of visceral leishmaniasis. Treatment of the diseases is limited by toxicity and parasite resistance to the drugs currently in use, validating the need to develop new leishmanicidal compounds. We evaluated the killing by the palladacycle complex DPPE 1.2 of *Leishmania (Leishmania) amazonensis*, an agent of human cutaneous leishmaniasis in the Amazon region, Brazil. DPPE 1.2 destroyed promastigotes of *L. (L.) amazonensis in vitro* at nanomolar concentrations, whereas intracellular amastigotes were killed at drug concentrations 10-fold less toxic than those displayed to macrophages. *L. (L.) amazonensis*-infected BALB/c mice treated by intralesional injection of DPPE 1.2 exhibited a significant decrease of foot lesion sizes and a 97% reduction of parasite burdens when compared to untreated controls. Additional experiments indicated the inhibition of the cathepsin B activity of *L. (L.) amazonensis* amastigotes by DPPE 1.2. Further studies are needed to explore the potential of DPPE 1.2 as an additional option for the chemotherapy of leishmaniasis.

## Introduction

Protozoan parasites of the *Leishmania* genus induce cutaneous, mucocutaneous and visceral diseases in man and animals. According to the World Health Organization, about 1.5 million of new human cases of cutaneous leishmaniasis and 500,000 of visceral leishmaniasis are registered annually [1]. *Leishmania (Leishmania) amazonensis*, one of the causative agents of human cutaneous leishmaniasis in the Amazon region, Brazil, is associated with both the simple and diffuse forms of the disease [2]. The first-line drugs used for treatment of leishmaniasis are pentavalent antimonial compounds, while amphotericin B and pentamidine are used as the second-line chemotherapy. However, the use of these compounds is limited by toxicity to the host and the development of resistance by the parasites [3], [4]. Thus, the development of new leishmanicidal drugs is an important goal and several compounds including synthetic, natural products extracted from plants and marine sources have shown different degrees of efficacy in the treatment of experimental leishmaniasis [5]–[7]. The *in vitro* and *in vivo* demonstration that the viability of the *Leishmania* parasites is reduced by inhibitors of cysteine proteases [8]–[10] encouraged the use of virtual screening to identify additional inhibitors [11], [12]. The demonstration that antitumor drugs may also display antileishmanial activity has also stimulated the screening of these compounds *in vitro* and in clinical trials [13]. Cyclopalladated complexes have shown *in vitro* and *in vivo* antitumor activity and low toxicity in animals [14]–[16] and more recently one of them exhibited lethal effects on human leukaemia cells while was ineffective against normal human lymphocytes [17]. The leishmanicidal and tripanocidal activity of cyclopalladated complexes has also been demonstrated [18]–[20]. Furthermore, there is evidence that palladacycle complexes may destroy tumoral cells by inhibition of cathepsin B activity and their inhibitory effect on *Leishmania* cysteine proteases *in vitro* was also demonstrated [18], [21]. The present study describes the effect of one palladacycle compound called DPPE 1.2 on promastigotes, intracellular amastigotes and cutaneous lesions in mice infected with *L. (L.) amazonensis*.

## Methods

### Animals

Eight-week-old female Golden hamsters were obtained from breeding stocks maintained at the Universidade de Campinas (São Paulo, Brazil) and female BALB/c mice 6 to 8 weeks old were acquired from Universidade Federal de São Paulo (São Paulo, Brazil). This study was carried out in strict accordance with the recommendations in the Guide for the Care and Use of Laboratory Animals of the Brazilian National Council of Animal Experimentation (http://www.cobea.org.br). The protocol was approved by the Committee on the Ethics of Animal Experiments of the Institutional Animal Care and Use Committee at the Federal University of São Paulo (Id # CEP 1844/08).

### Parasites

The *L. (L.) amazonensis* strain used (MHOM/BR/1973/M2269) was kindly provided by Dr. Jeffrey J. Shaw, Instituto Evandro Chagas, Belém, Pará, Brazil and maintained as amastigotes by inoculation into footpads of Golden hamsters every 4 to 6 weeks. Amastigote suspensions were prepared by homogenization of excised lesions, disruption by four passages through 22-gauge needles, and centrifugation at 250×*g* for 10 min; the resulting supernatant was centrifuged at 1,400×g for 10 min, and the pellet was resuspended in RPMI 1640. The suspension was kept under agitation for 4 h at room temperature and centrifuged at 250×g for 10 min. The final pellet contained purified amastigotes which were essentially free of contamination by other cells [22].

*L. (L.) amazonensis* promastigotes were grown at 26°C in 199 medium (Gibco) supplemented with 4.2 mM sodium bicarbonate, 4.2 mM HEPES, 1 mM adenine, 5 µg/ml hemin (bovine type I) (Sigma, St Louis, MO, USA) and 10% fetal calf serum (FCS) (Cultilab, SP, Brazil).

### Biphosphinic palladacycle complex [Pd(C2, N-*S*(*-*)DMPA)(DPPE)]Cl (DPPE 1.2)

**Figure 1:**
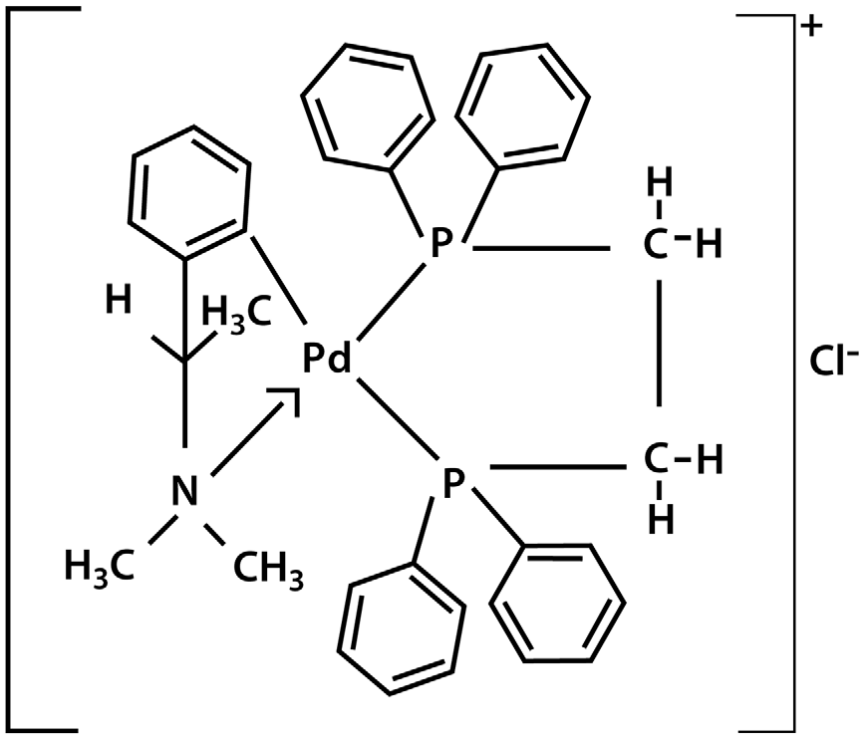
Structure of the DPPE 1.2 compound [Pd(C2, N-*S*(*-*)DMPA)(DPPE)]Cl.

The palladacycle compound DPPE 1.2 (Figure 1) was obtained from N,N-dimethyl-1-phenethylamine (DMPA), complexed to 1,2-ethane-bis (diphenylphosphine) (DPPE) ligand and synthesized as previously described [16]. Stock solutions at 1.45 mM were prepared in dimethylsulfoxide (DMSO); for *in vitro* use, the drug was diluted to the appropriate concentration in cell culture medium, and for *in vivo* injections the stock was diluted in PBS.

### Effect of DPPE 1.2 on *L. (L.) amazonensis* promastigotes and intracellular amastigotes

The promastigote cultures at 1×10^6^ parasites/ml were kept in 199 culture medium as described above containing between 1.25 nM and 150 nM of DPPE 1.2. Parasites were counted daily in a Neubauer chamber for three days. The leishmanicidal effect of DPPE 1.2 on intracellular amastigotes was evaluated in mouse bone marrow derived macrophages infected with *L. (L.) amazonensis*. Bone marrow-derived macrophages were generated from bone marrow stem cells isolated from BALB/c mice [23]. Cells were counted, added (8×10^5^) and cultured on glass coverslips inserted in 24-well tissue culture plates containing RPMI 1640 medium buffered with 15 mM of HEPES, 20 mM of sodium bicarbonate and supplemented with 1 mM L-glutamine, 20% of fetal calf serum (FCS) and 30% L929 cell conditioned medium (LCCM). Cultures were kept at 37°C in an atmosphere of air/CO_2_ (95/5%). After 5 days, the medium was changed for RPMI containing 10% of FCS and macrophages were infected at a multiplicity of 2 amastigotes per macrophage. After 24 h, infected cultures were treated with different drug concentrations (150 to 500 nM) for 3 days. The coverslips were fixed with methanol, stained with hematoxylin-eosin (HE) and intracellular amastigotes were counted. Results are expressed by the infection index, obtained by multiplying the percentage of infected macrophages by the average number of amastigotes per macrophage. At least 200 macrophages were scored in each 3 coverslips. Amphotericin B (Sigma-Aldrich, St Louis, MO, USA) and Glucantime (Sanofi-Aventis, Brazil, 300 mg/ml, 81 mg/ml Sb^V^) were used as standard drugs for treatment of *L. (L.) amazonensis* promastigotes and intracellular amastigotes, respectively.

### Cytotoxicity assays

DPPE 1.2 cytotoxicity to macrophages was tested by a MTT micromethod described previously [24] after incubation of bone marrow derived macrophages with 150 to 2,000 nM of DPPE 1.2 for 3 days. Macrophages were also incubated with the highest concentration of DMSO used for DPPE 1.2 solubilization (0.04%). The formation of formazan was measured by adding 3-(4,5-dimethylthiazol-2-yl)-2,5-diphenyltetrazolium bromide (MTT; Molecular Probes, Eugene, OR, USA) 0.5 mg/ml and incubation of the cultures at 37°C in the dark. After 4 h the medium was removed, 200 µl of DMSO was added per well and the absorbance was measured using an ELISA reader at 540 nm (Labsystems Multiskan).

### Antileishmanial *in vivo* assays

For evaluation of *in vivo* leishmanicidal activity of DPPE 1.2 female BALB/c mice 6 to 8 weeks-old were infected subcutaneously at the right hind-foot with 1×10^5^ *L. (L.) amazonensis* amastigotes. Fifteen days after infection, the animals were randomly separated in 3 groups of 12 mice each. Treated animals received in the foot lesions every other day doses of 60 mg/kg/day (16.8 mg [Sb^v^]/kg/day) of Glucantime for 1 month (total of 900 mg/kg–252 mg [Sb^v^]/kg/day) or doses of 320 µg/kg/day of DPPE 1.2 (total of 4.8 mg/kg). Stock solutions of DPPE 1.2 were prepared daily in PBS after solubilization in DMSO (final concentration of 0.1%). Control group received the same number of injections of PBS. Infection was monitored once a week by measuring the diameter of foot lesions with a dial caliper (Mitutoyo Corp., Japan). Parasite burden from infected feet was determined by a limiting dilution method, as previously described [25].

### Toxicity for mice assays

Serum concentrations of urea, creatinine, bilirubin and transaminases were determined in BALB/c mice at the end of treatment, using sets of commercial reagents (Doles Reagentes e Equipamentos para Laboratórios, Ltda, Brazil).

### Proteolytic activity

Proteolytic activity of *L. (L.) amazonensis* promastigotes and amastigotes was determined by zymography employing electrophoretic separation of parasite lysates under unheated and nonreduced conditions resolved on 10% acrylamide gels containing 0.1% copolymerized gelatin (Gibco-BRL) by low-voltage (50 V) electrophoresis [26]. Proteolytic activity in the gels was detected after 1 h of incubation, under agitation, in 0.1 M sodium acetate buffer, pH 5.0, containing 2.5% Triton X-100, followed by 2 h of incubation in the acetate buffer in the absence of Triton X-100 and Coomassie blue staining. Some gel strips after electrophoresis were incubated in buffer solutions in the presence of either protease inhibitor E-64 (trans-epox-isuccinil-L-leucinamide-(4-guanide-butane) or orthophenanthroline or DPPE 1.2. Molecular weight markers (Pharmacia LKB) were visible on the background of stained gelatin when used in a 5-fold excess.

### Inhibition of *L. (L.) amazonensis* cathepsins by DPPE 1.2

Cathepsin activities were monitored with the fluorogenic substrates Z-Phe-Arg-AMC (for all cathepsins), Z-Arg-Arg-AMC (for cathepsin B), and Z-Leu-Arg-AMC (for cathepsins K, V, and S) (commercially obtained from Sigma, St. Louis, MO, USA) using 1 µl of *L. (L.) amazonensis* amastigote cell lysate (1×10^9^ amastigotes disrupted in 200 µl PBS), 2 mM DTT (dithiothreitol), 1 ml of four-component buffer comprised of 25 mM acetic acid, 25 mM Mes (4-Morpholineethanesulfonic acid), 75 mM Tris, and 25 mM glycine, pH 5.0, 10 µM of each fluorogenic substrate and 50 µM of DPPE 1.2. The effect of DPPE 1.2 on the parasite enzyme activity was tested by incubation of the *L. (L.) amazonensis* lysate with DPPE 1.2 for 2 minutes in the buffer solution a 37°C; the fluorogenic substrate was then added and the fluorescence of the released fluorophore, 7-amino-4-methylcoumarine (AMC), was measured over time. The remaining enzyme activities were determined and expressed as a percentage of the activity of the control experiment. Parasite lysate was also incubated with 10 µM of the fluorogenic substrate Abz-Gly-Ile-Val-Arg-Ala-Lys(Dnp)-OH (Sigma, St. Louis, MO, USA), specific for cathepsin B [27], in the presence of either increasing concentrations of DPPE 1.2 or CA074, a specific inhibitor of cathepsin B. The cathepsin activity was monitored spectrofluorometrically using the fluorogenic substrates on a Hitachi F-2000 spectrofluorometer equipped with a thermostated cell holder. The fluorescence excitation (λEx) and emission (λEm) wavelengths, for the fluorescence of AMC, were set at 380 nm and 460 nm, respectively, while the parameters for the fluorescence of Abz-peptide fragments resulting from the Abz-Gly-Ile-Val-Arg-Ala-Lys(Dnp)-OH hydrolysis were set at λEx = 320 and λEm = 420 nm.

### Statistical analysis

To determine the statistical differences between groups ANOVA and Student's *t* test were used and *P* values<0.05 or lower were considered statistically significant. IC50 and CC50 values were determined by GraphPad Prism, version 5.0.

## Results

### Inhibitory activity of DPPE 1.2 on growth of *L. (L.) amazonensis* promastigotes

Axenic cultures of *L. (L.) amazonensis* promastigotes were grown in the presence of 1.25 to 150 nM of DPPE 1.2. Significant inhibition of parasite growth was detected after 2 and 3 days of treatment with 1.25 to 25 nM of DPPE 1.2. At 75 and 150 nM the drug inhibited 84% and 96%, respectively, of parasite growth 1 day after treatment and nearly 100% of promastigotes were killed after 2 and 3 days in the presence of these concentrations of DPPE 1.2. A growth curve similar to control was observed when *L. (L.) amazonensis* promastigotes were cultured in the presence of the highest concentration of DMSO used for DPPE 1.2 solubilization (0.04%). As a control drug parasites were grown in the presence of amphotericin B. After 72 of incubation, the IC50 values for both drugs were determined (Table 1).

**Table 1:** *In vitro* activity of DPPE 1.2 and amphotericin B on *L. (L.) amazonensis* promastigotes.

Drug	IC50 (nM)^a^	CI 95%^b^
DPPE 1.2	2.13	2.02–2.24
Amphotericin B	15.58	9.1–22.6

a Values were calculated after 72 h of incubation with the drugs and are representative of three independent experiments.

b 95% confidence interval.

### Leishmanicidal activity of DPPE 1.2 on *L. (L.) amazonensis*-infected macrophages

**Figure 2:**
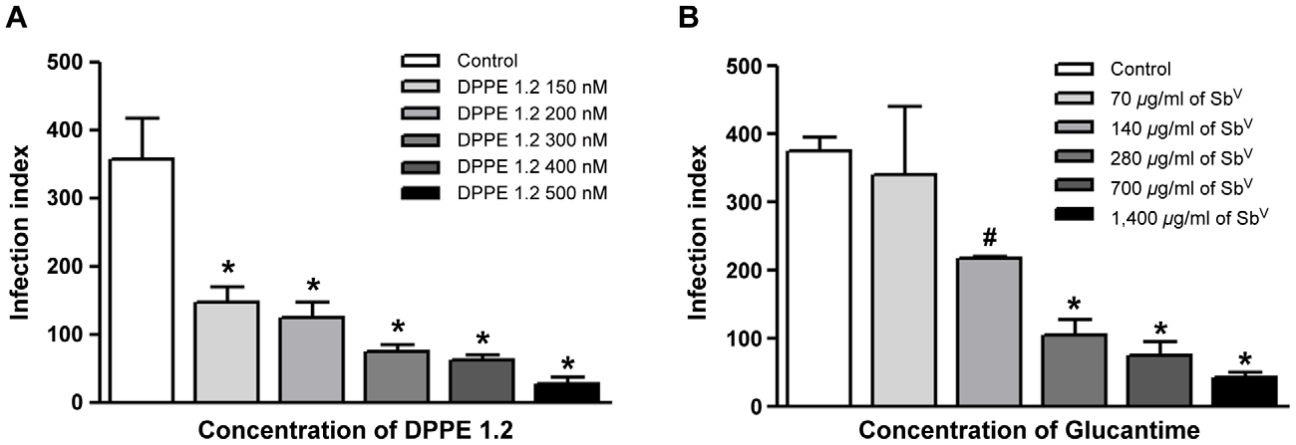
Effect of DPPE 1.2 and Glucantime on intracellular *L. (L.) amazonensis* amastigotes. Macrophages were infected with amastigotes of *L. (L.) amazonensis* for 24 h at 37°C, the drugs were added and infection index was calculated after 72 h of treatment. ^*^ *P*<0.001 and ^#^ *P*<0.05 compared to control.

Treatment with DPPE 1.2 resulted in a significant, dose-dependent decrease in infection index of *L. (L.) amazonensis*-infected macrophages with an inhibition of 92% for 500 nM of DPPE 1.2 (IC50 of 128.35 nM; 95% confidence limits, 111.2–164.2 nM) (Figure 2A). Infected cultures were also treated with the highest concentration of DMSO used for DPPE 1.2 solubilization (0.04%); these concentrations did not reduce the viability or the infection of macrophages (data not shown). The cytotoxicity of DPPE 1.2 on macrophages was evaluated by the MTT method and the CC50 was determined (1,267 nM; 95% confidence limits, 1,15–1,52 nM).

Figure 2B). The IC50 value expressed as µg/ml of pentavalent antimony [Sb^v^] was 178.5 µg/ml (95% confidence limits, 108.1–294.6 µg/ml) and treatment with the drug at this concentration resulted in 40% of macrophage toxicity (data not shown). The calculated CC50 for Glucantime expressed as µg/ml of pentavalent antimony [Sb^v^] was 266.3 µg/ml (95% confidence limits, 252.2–290.3 µg/ml).

### Effect of DPPE 1.2 on BALB/c mice infected with *L. (L.) amazonensis*

**Figure 3:**
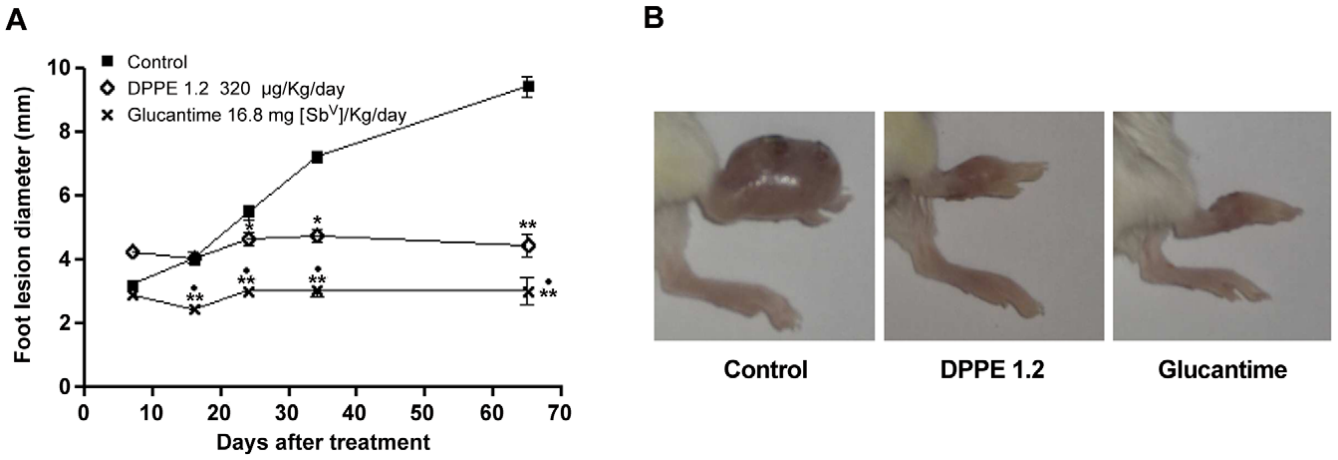
Effect of DPPE 1.2 on BALB/c mice infected with *L. (L.) amazonensis*. A -Development of foot lesions in *L. (L.) amazonensis*-infected BALB/c mice treated with DPPE 1.2. The treatment was started 15 days after infection and continued for 5 weeks. Data points represent the average measurements for 3 groups of twelve mice each; B - Macroscopical evaluation of lesions in untreated (left column), DPPE 1.2-treated mice (medium column), and Glucantime-treated mice (right column) 60 days post-infection. ^*^ *P*<0.01 and ^**^ *P*<0.001 compared to untreated animals; ^#^ *P*<0.001 compared to animals treated with DPPE 1.2. Data are representative of three independent experiments.

BALB/c mice infected with *L. (L.) amazonensis* were treated every other day with 320 µg/kg/day of DPPE 1.2 for 1 month injected in foot lesions. As can be observed in Figure 3, starting from 24 days of treatment the animals which received DPPE 1.2 showed a significant decrease of foot lesion size compared to controls. Starting from 16 days of treatment, the animals that received Glucantime also exhibited significantly smaller foot lesions compared to untreated control, as well as to animals treated with DPPE 1.2.

**Figure 4:**
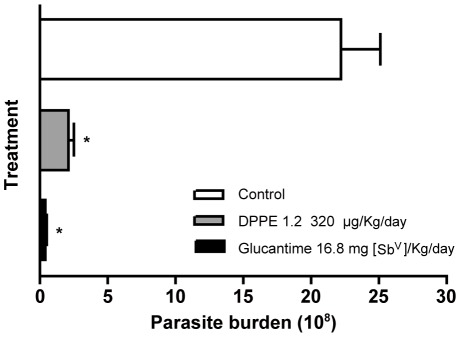
Parasite load in foot lesions of *L. (L.) amazonenis*-infected BALB/c mice treated with DPPE 1.2. Number of parasites recovered by limiting dilution from *L. (L.) amazonensis*-infected mice treated with DPPE 1.2. Parasites were quantified one month after interruption of treatment. ^*^ *P*<0.001.

Parasite load was also evaluated by limiting dilution in foot lesions of BALB/c mice one month after end of the treatment with either DPPE 1.2 or Glucantime. Figure 4 shows that BALB/c mice treated with either DPPE 1.2 or Glucantime displayed a reduction of parasite load of 97% and 99%, respectively, compared to untreated animals.

To evaluate hepato and nephrotoxicity of DPPE 1.2 serum levels of transaminases, urea and creatinine were determined. No statistically significant alterations were detected between groups (data not shown).

### Effect of DPPE 1.2 on proteolytic activity of *L. (L.) amazonensis*

**Figure 5:**
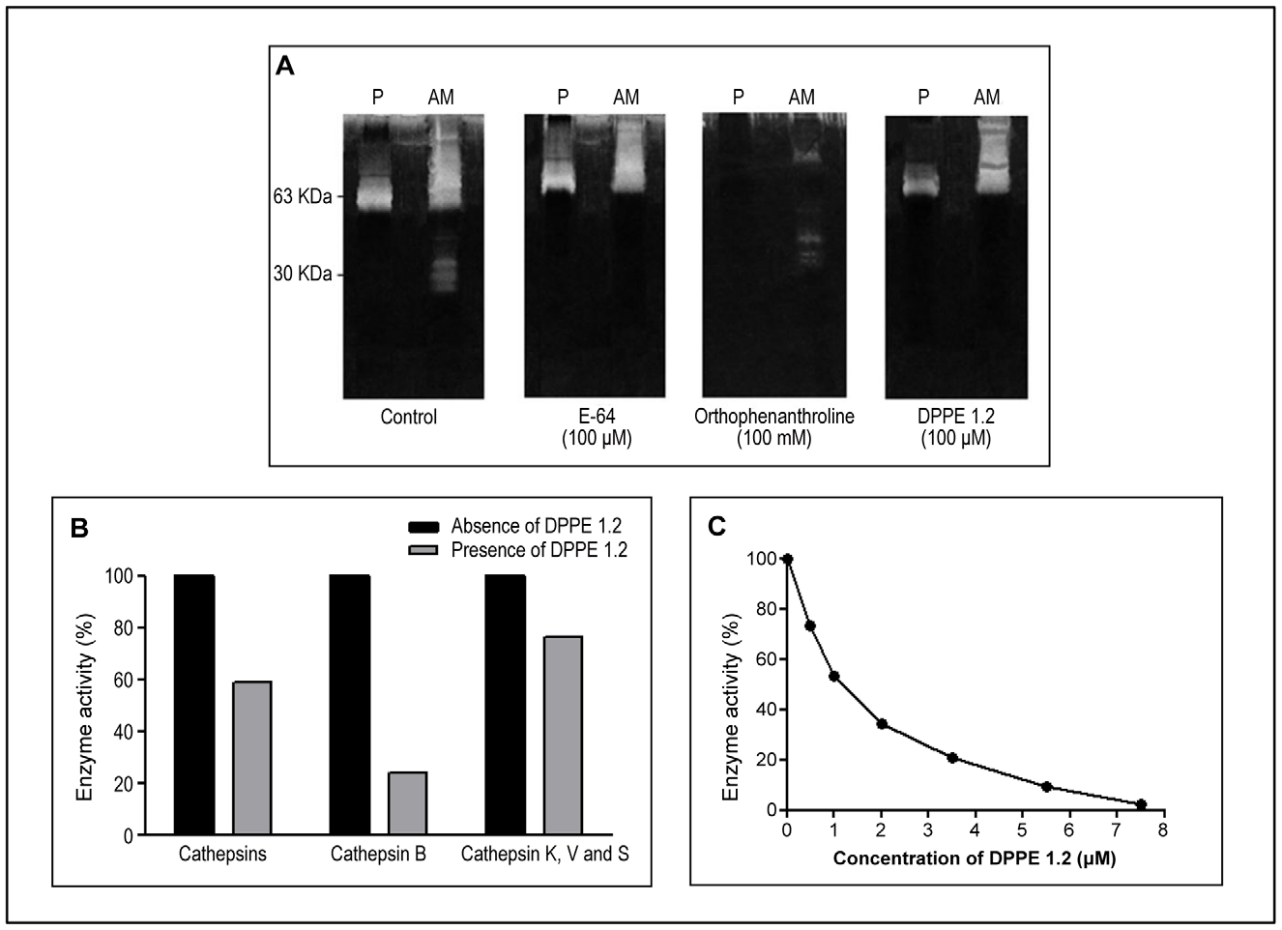
Effect of DPPE 1.2 on proteolytic activity of *L. (L.) amazonensis*. A - Extracts of promastigotes (P) and amastigotes (AM) of *L. (L.) amazonensis* were separated by SDS-PAGE on a 10% acrylamide gel containing 0.1% gelatin under nonreducing conditions. Numbers at left indicate apparent molecular masses in kilodaltons. B - Fluorogenic substrates with different specificities for cathepsin-like proteases were incubated with extracts of *L. (L.) amazonensis* amastigotes in absence or presence of DPPE 1.2, as indicated. C – Fluorogenic substrate specific for cathepsin B-like proteases was incubated with extracts of *L. (L.) amazonensis* in presence of increasing concentrations of DPPE 1.2.

Parasite proteolytic activity was determined by zymography after electrophoresis of *L. (L.) amazonensis* extracts in SDS-PAGE with gelatin coupled gels. Figure 5A shows that most of the proteolytic activity of *L. (L.) amazonensis* promastigotes that migrates as a 60 kDa band was abolished in the presence of orthophenanthroline, while DPPE 1.2 did not show any effect on this activity. On the other hand, amastigotes displayed a strong activity at a molecular mass of 30–35 kDa that was totally inhibited by either DPPE 1.2 or E-64, indicating that DPPE 1.2 inhibits cysteine protease activity of *L. (L.) amazonensis* amastigotes.

A spectrofluorometric assay using specific substrates for cathepsins in the presence of DPPE 1.2 was also carried out. Figure 5B shows that the *L. (L.) amazonensis* amastigote extract exhibited high hydrolytic activity on all substrates tested. Although DPPE 1.2 inhibited the enzymatic activity on all substrates, a significantly higher reduction on cathepsin B activity could be observed in the presence of this palladacycle complex (75%). Figure 5C shows that the activity of *L. (L.) amazonensis* extract on a most specific substrate for cathepsin B was significantly inhibited either by DPPE 1.2 or CA074 (data not shown). The calculated IC50 values for DPPE 1.2 and CA074 were not significantly different (2.25±0.11 µM and 0.7±0.08 µM, respectively), strongly suggesting that DPPE 1.2 inhibits *L. (L.) amazonensis* cathepsin B.

## Discussion

The present results document the leishmanicidal effect of the palladacycle complex DPPE 1.2 on *L. (L.) amazonensis*. This compound destroyed *L. (L.) amazonensis* promastigotes at very low concentrations. Extension of this study to *L. (L.) amazonensis*-infected macrophages also showed an effective leishmanicidal activity of DPPE 1.2 against amastigotes, whereas the drug displayed 10-fold less toxicity to macrophages. Although similar leishmanicidal effect was observed with Glucantime, significantly higher concentrations of this antimonial were necessary to destroy *L. (L.) amazonensis* amastigotes. The leishmanicidal activity of DPPE 1.2 is comparable to that obtained with several compounds tested against *L. (L.) amazonensis* like mesoionic salt derivatives, primary *S*-nitrosothiols, aureobasidin A, julocrotine, tamoxifen, elatol [28]–[33]. However, higher concentrations of these compounds were used to destroy *L. (L.) amazonensis*, whereas an effective leishmanicidal effect was observed with DPPE 1.2 at nanomolar range. The leishmanicidal activity of metal complexes of gold, platinum, iridium, rhodium and osmium has also been investigated. However, most of them destroyed only *L. (L.) donovani* promastigotes, while few reduced the parasitism in infected animals [34]–[36], impairing the comparison with DPPE 1.2 data. Among other palladium complexes previously tested against *Leishmania* only one was an effective inhibitor of promastigote growth, while none of them reduced the intracellular amastigote burden [18].

The leishmanicidal effect of DPPE 1.2 was also demonstrated *in vivo*. Although the reduction of parasite load in foot lesions of *L. (L.) amazonensis*-infected mice treated with DPPE 1.2 was similar to that obtained with Glucantime, this antimonial compound was used in 200 times higher concentration. Treatment with DPPE 1.2 led to a significant reduction of parasite load in foot lesions (97%), but did not result in sterile cure in infected mice. However, it is important to emphasize that the BALB/c strain is highly susceptible to *L. (L.) amazonensis* infection. These mice develop a gradual increase of foot lesions characterized by a large infiltrate of macrophages harboring a high number of amastigotes, thus mimicking the anergic form of diffuse cutaneous leishmaniasis caused by *L. (L.) amazonensis* [2]. The apparent lack of toxicity of DPPE 1.2 to BALB/c mice was demonstrated by hepatic and renal function assays after treatment with the drug, corroborating data that showed the low toxicity of palladacycle complexes in the treatment of mice against tumor cells [16]. More recently, the high selectivity index of one cyclopalladacycle complex with trypanocidal activity was also demonstrated, suggesting the use of this compound for treatment of Chagas' disease [19].

As reported in the literature, the antitumor property of palladacycle complexes can be attributed, at least in part, to their inhibitory activity on the cysteine protease cathepsin B [21]. This information led us to test for the possible effect of DPPE 1.2 on *L. (L.) amazonensis* protease activity. We showed that DPPE 1.2 did not inhibit the activity of the metalloprotease gp63, the major surface protein of *Leishmania* promastigotes [37]. On the other hand, the high cysteine protease activity expressed in *L. (L.) amazonensis* amastigotes was inhibited by DPPE 1.2 and the most significant inhibition was observed on the cathepsin B activity. However, the drug did not affect the cysteine proteinase activity of mouse macrophages (data not shown). Several studies demonstrated the involvement of cathepsin L-like (cpL) and cathepsin B-like (cpB) in *Leishmania* growth and virulence *in vitro* and *in vivo* [38]–[40]. Furthermore, cysteine proteinase inhibitors have been reported to kill *Leishmania in vitro* and *in vivo* [8]–[10]. In the present study we show that *in vitro* DPPE 1.2 inhibited *L. (L.) amazonensis* cathepsin B at higher concentrations than those necessary to kill *L. (L.) amazonensi*s. These findings argue against a relationship between the leishmanicidal effect of DPPE 1.2 and the inhibition of *L. (L.) amazonensis* cathepsin B and suggest that other relevant targets may account for the leishmanicidal effect of the drug. Palladacycle complexes have been associated with organelle-specific effects in tumor cells such as the lysosomal and mitochondrial permeabilization that can trigger apoptosis [41], [42]. The induction of *L. (L.) amazonensis* apoptosis by DPPE 1.2 has not been investigated.

*Leishmania* killing is associated to macrophage activation by IFN-γ and TNF-α and the production of nitric oxide [43], whereas TGF-β is an immunosupressor cytokine known to exacerbate visceral and cutaneous leishmaniasis [44]–[47]. Interestingly, *Leishmania* cathepsin B is involved in the conversion of latent TGF-β to its biologically active form [48]. Since we have shown here that DPPE 1.2 inhibits parasite cathepsin B, killing of *L. (L.) amazonensis* in infected mice treated with the drug may be associated to protective responses arising from lower expression of the active form of TGF-β. This possibility is now under investigation.

In conclusion, the effectiveness of DPPE 1.2 in destroying *L. (L.) amazonensis in vitro* and by intralesional administration *in vivo* at concentrations non toxic to the host support further studies of the leishmanicidal activity of the palladacycle as an additional choice to available chemotherapies.
